# State Ensemble Energy Recognition (SEER): A Hybrid Gas-Phase Molecular Charge State Predictor

**DOI:** 10.1021/acs.jcim.5c00980

**Published:** 2025-07-08

**Authors:** Mithony Keng, Kenneth M. Merz

**Affiliations:** † Department of Chemistry, 3078 Michigan State University, East Lansing, Michigan 48824, United States; ‡ Department of Biochemistry and Molecular Biology, 3078 Michigan State University, East Lansing, Michigan 48824, United States

## Abstract

Accurately resolving a three-dimensional structure that corresponds to an experimental mass spectrometry (MS) result is valuable for outcomes such as improved analyte identification, determination of physiochemical properties relating to conformation, analyte impurity testing, and drug chemical integrity analysis. Computational approaches utilizing charge state modeling, conformational sampling, quantum mechanical optimizations, relative energy scoring, and computed ion-neutral collision cross sections (CCS) have historically achieved success at assigning equilibrium structures to ion-mobility MS-derived CCS values. Despite this positive status, there remains a lack of new computational software to achieve higher throughput when modeling large systems. A major adverse impact on computational cost is the general increase in titratable sites with molecular size, which then warrants additional protonation/deprotonation models in order to ensure that the correct charge state is captured. Here, we introduce a user-friendly machine learning program called SEER (**S**tate **E**nsemble **E**nergy **R**ecognition) to accurately and efficiently predict the equilibrium charge states of MS-relevant ions. We report that for all systems within the test set, SEER successfully captured the lowest relative energy minimum charge states within its top two predicted candidates from an overall average number of ∼ seven titratable sites. Furthermore, the density functional theory optimized geometries for SEER assigned charge states produced CCS experimental errors that are within the acceptable threshold (i.e., ≤3% error) set for this work. The benchmark study compared SEER to two well-established charge state prediction software packages CREST and Epik classic and found that SEER is either on par or better at consistently locating the correct charge states for the test set with competitive efficiency. SEER requires no additional user programming and is readily accessible through the Google Colab platform at https://github.com/mitkeng/SEER.

## Graphical abstract

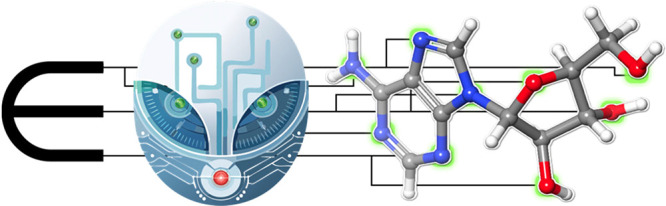

## Introduction

Accurately resolving a three-dimensional structure that corresponds to an experimental mass spectrometry (MS) result is valuable for such goals as analyte identification, determination of gas-phase thermodynamic properties, analyte impurity testing, and drug chemical integrity analysis. Increasingly, ion mobility mass spectrometry (IM-MS) has been applied to bring about greater structural distinction and information to analyte identification, which is especially beneficial for separating analytes with very similar or equal mass (i.e., isobars).^1–4^ This is accomplished through the differentiable ion mobility observed in an IM-MS drift region as a result of analyte size-dependent ion-neutral collisions. That is, a larger ion tends to experience more frequent collisions and greater momentum transfer with a neutral buffer gas (e.g., nitrogen or helium) than a smaller-sized ion, which ultimately leads to different arrival times at a detector. Conversely, the relative size of an ion can in turn be determined by calculating its collision cross section (CCS) using the Mason–Schamp equation and ion mobility data.^5^ From CCS values, resolving finer details such as molecular conformation or a protonation/deprotonation pattern on an ion requires the use of computational approaches.

It has become a standard practice in chemical structure prediction regimens to synergically incorporate charge state modeling, conformational sampling, quantum mechanical (QM) level geometry optimization, relative energy scoring, and computed CCS values to successfully locate valid gas-phase equilibrium geometries.^6–11^ The most computationally expensive step by far within such a workflow, and essentially the bottleneck, is the QM processing phase. To improve efficiency, lower-level electronic structure theory^12,13^ (i.e., Hartree–Fock and semiempirical), small basis sets^14–16^ (e.g., STO-3G and 3-21G), or neural network potentials (e.g., ANAKIN-ME) are options that have been shown to achieve reasonable results for predicting equilibrium geometries for small systems. However, from our previous studies^8,10,11^ in structure predictions, we found that density functional theory (DFT) using the B3LYP functional with dispersion correction at a minimum basis set size of 6-31G­(d,p) is effective at generalizability (i.e., size and molecular class) and accuracy, as demonstrated through good agreement with experiment. Thus, rather than undermining quality with less precise QM methods or alternatives for a minimal speedup, a best practice approach to improve efficiency and enhance throughput is to reduce any unnecessary work that goes into the QM step.

From experience, charge state modeling is the most manual time-consuming task and is the most time-consuming step. Despite this, a thorough charge site enumeration to arrive at a ground truth charge state (atomic site bearing either the negative charge or positive charge) is required for good agreement with the experiment, regardless of how thoroughly a conformational space is sampled. Other than lipids, titratable sites generally increase somewhat linearly with molecular size for biomolecules and small molecules, making the charge site enumeration task more taxing as well as leading to a larger volume downstream for QM calculations. For the common IM-MS ion charge modes [M – H]– and [M + H]+, a manual deprotonation/protonation process informed by using p*K*_a_ or proton affinity information is typically used. However, experimental p*K*_a_ lacks the relevant MS solvent profile used in electrospray ionization (ESI)^17^ experiments (e.g., acetonitrile, ethanol, tetrahydrofuran, dichloromethane) and is a property of solution phases rather than the gas phase; likewise, experimental information on gas-phase proton affinities is limited and, therefore, is not particularly suitable for routine prediction of gas-phase charge states that originate in solution for complex molecules.

Some currently well-established software packages that have been used to predict protonation/deprotonation sites include CREST^18^ (open-source), Dimorphite-DL^19^ (open-source), and Schrödinger’s Epik^20^ (commercial). Both Dimorphite-DL and Epik are p*K*_a_ based methods for protomer (protonation state tautomer) prediction, whereas CREST employs ensemble sampling coupled to gas-phase geometry optimization and energy calculation using the semiempirical QM method GFN2-xTB to find stable protomers. All three software packages can also automatically generate an appropriate [M – H]– or [M + H]+ 3D structure, which eliminates the need to manually remove or add a proton. However, these methods, along with others, are not specifically parametrized for or designed to target typical IM-MS gas-phase experiments, which environmental conditions can foster incidences of charge/proton migration^21–24^ (i.e., prototropic tautomerism).

Here, we introduce a hybrid machine learning program called SEER (**S**tate **E**nsemble **E**nergy **R**ecognition) for the prediction of gas-phase molecular protonation states that are relevant to mass spectrometry. SEER employs a regression model trained on DFT ground truth and, also, the ANAKIN-ME^25^ (ANI) neural network potential for a two-factor screening of enumerated charge sites. The functionalities that a user can expect from SEER are ranking all viable titratable sites, unambiguous assignment of an equilibrium charge state(s), geometry optimization, automatic generation of a deprotonated or protonated form of an input structure, and, finally, compute relative energies and preliminary gas-phase mole fractions of protonation/deprotonation models. A programmatic scheme of SEER’s workflow is shown in Figure 1. In this manuscript, we will go through the development and validation process for SEER that starts from the procurement of experimentally accurate molecular geometries for training, feature selection and engineering, ML model selection to software performance on unseen test systems, and benchmarking.

**Figure 1:**
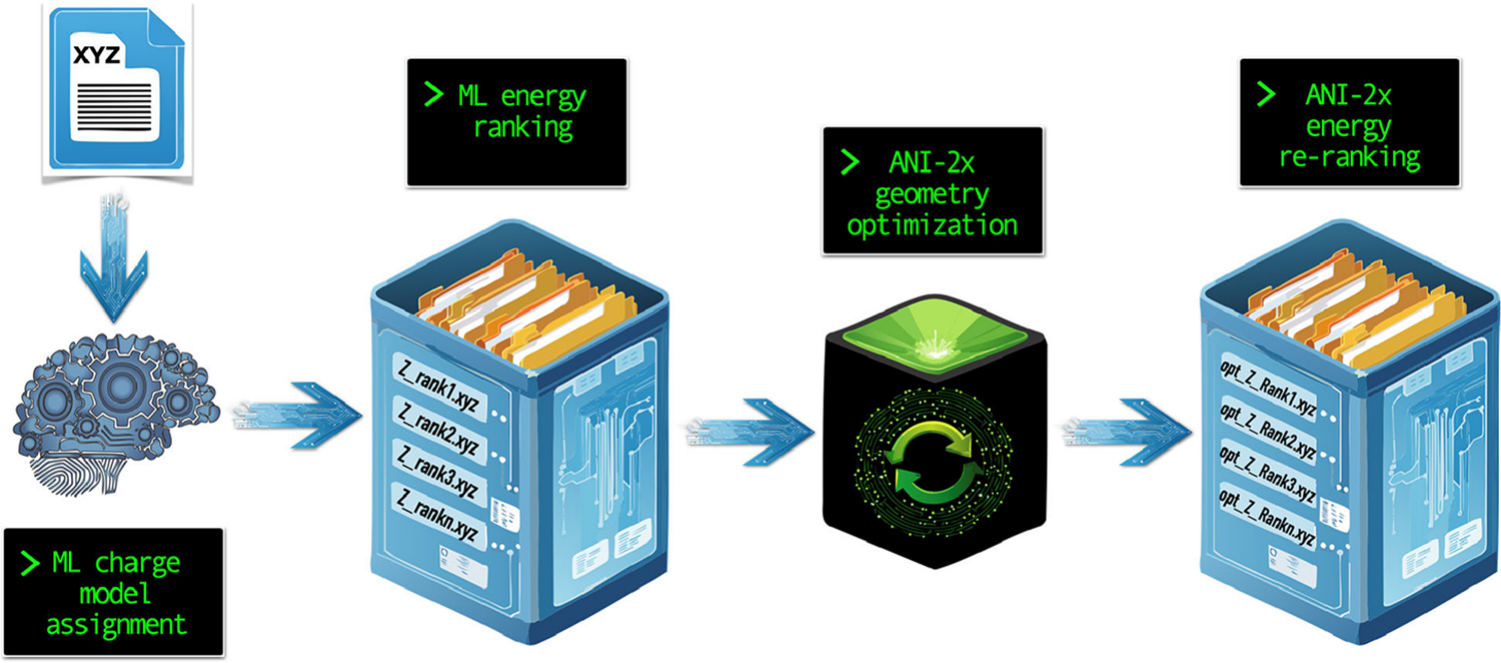
Programmatic scheme of the hallmark steps going from input to output of results output. *Z* represents a protonation/deprotonation model and *opt* is for optimized. Initial and final protonation/deprotonation model rankings are designated with a lower “r” and a capital “R”, respectively.

## Computational Methods

### Molecular Geometries Used in Training

For each chemical system used in the training data set, an ensemble of conformations for commonly titratable atoms was generated. All conformations were generated and clustered using Schrödinger’s ConfGen^26^ and the open-source Autograph,^27^ respectively. Geometry optimization and single-point energy calculation were carried out on the ensembles at the D3BJ-B3LYP/6-31G­(d,p) and D3BJ-B3LYP/6-31+G­(d,p) level for the protonated (i.e., [M + H]+) and deprotonated (i.e., [M – H]−) ion modes, respectively, through the Gaussian 16 software package.^28^ A protonation/deprotonation model that produced the lowest relative energy across all models for a system was chosen as the equilibrium charge state for that system. CCS values were computed for the optimized geometries using the open-source software HPCCS.^29^ We judged the accuracy of the predicted candidate charge state structures for each system by comparing the computed CCS with a reference experimental CCS. Only ensembles of protonation/deprotonation models (all models were included and not just the candidate charge state) for systems that had CCS errors of ≤3% (the 3% is to account for the uncertainty in an IM-MS experiment) were included in the training data set. A simple schematic of the workflow that is described here is displayed in Figure S1 of the Supporting Information.

### Feature Selection and Engineering

A total of 10 features are used to construct the training data set for the first pass regression model: (1) atomic polarizability of the charge bearing atom (CBA), (2) CBA distance from center of mass (COM), (3) CBA distance from center of electronegativity (COE), (4) distance between COM and COE, (5) interaction angle, (6) molecular surface area (MSA), (7) total oxygen, (8) total nitrogen, (9) total halogen, and (10) total “othergen” (i.e., sulfur, phosphorus, and selenium). COM and MSA were calculated using ASE (Atomic Simulation Environment)^30^ and the PyMOL^31^ API. COE is similar to COM except that mass is substituted for an electronegativity value and can be calculated using

$$\mathrm{COE}=\sum_{i=1}^{n}\frac{e_{i}r_{i}}{E}$$ 1

where *e* is the electronegativity for atom *i*, *r* is the Cartesian coordinate of atom *i,* and *E* is the sum of the electronegativity values for all atoms in a molecule. The interaction angle is the angle formed between the positions of COM, COE, and CBA. For each system, these features together tether a unique 3D conformational identity to a relative energy (RE) label that is calculated across all modeled charge states. Intuitively, the features used are intended to help the model differentiate protonation/deprotonation behaviors by tethering favorable titratable sites (i.e., via a label) to chemically specific steric and electronic characteristics. For example, the COE can provide correlation between the site of molecular electron density and the preferred site for proton attraction (i.e., protonation target). The importance of these features is quantified by evaluating the ML model mean increase RMSE (MIR) as a consequence of each feature’s successive elimination from the training data (for MIR results for top features, see Table S1 in the Supporting Information).

### Implementation of Machine Learning Models

The first pass screening and initial ranking of charge sites or titratable atoms (i.e., N or O) are accomplished using the YDF (Yggdrasil Decision Forests)^32^ gradient boosted tree (GBT) learner model, which is a supervised learning technique that employs a gradient of ensemble prediction models for decision-making. We trained the model on 1176 examples for the positive mode, [M + H]+, and ∼1300 examples for the negative mode, [M – H]–. A complete hyperparameter setting is tabulated in Table S2 in the Supporting Information. The SEER algorithm first uses the gradient boosted model to predict relative energies via regression. Thus, the models (protonated or deprotonated) are ranked according to the RE values, with the lowest RE (most stable) having a *Rank* 1. Using eq 2, the predicted RE results and the current root-mean-square error (RMSE) performance of the GBT model during training were used to calculate the acceptance capacity, ∧, to determine the number of original protonation/deprotonation models to move forward to the next process.

$$\wedge =\frac{1}{3}\mathrm{RMSE}+\frac{{\mathrm{RE}}_{\min}}{\sigma +S}$$ 2

where RE_min_ is the minimum relative energy predicted; σ and *S* are the standard deviation and the variance, respectively, for the predicted RE values for the protonation/deprotonation models of a system; and the 1/3 prefactor is a heuristically determined value that is used to add more generalization to the selectiveness of ∧. The utility of ∧ is to reduce false positives from entering the second screening process while also safeguarding against the loss of true positives.

Next, the ranked protonation/deprotonation models that made it through the first pass were treated with a “soft” geometry optimization (optimization step is set to 50) using the neural network potential model ANI-2x.^33^ This level of optimization was chosen to prevent the occurrence of structural artifacts while still calculating reasonable RE. Although ANI-2x only supports the atom types H, C, N, O, F, Cl, and S, we enabled SEER to carry out surrogate optimization for the additional atom types P, Se, Br, and I by switching atom types. For example, an unsupported halogen atom is switched for Cl (closest supported halogen neighbor); similarly, P or Se is switched for S. After geometry optimization, we switch any substituted atom back to its original atom type. Overall, we believe that this is an acceptable approach given the geometries generated by SEER at this step are intermediate structures and not the final global minima. Nevertheless, we randomly tested biotin and moclobemide to compare the charge sites generated using this surrogacy approach. The results shown in Figure 2 establish that the equilibrium charge sites for biotin and moclobemide for S ↔ Se and Br ↔ Cl, respectively, are conserved, and we therefore satisfactorily show that our approximation scheme is viable.

**Figure 2:**
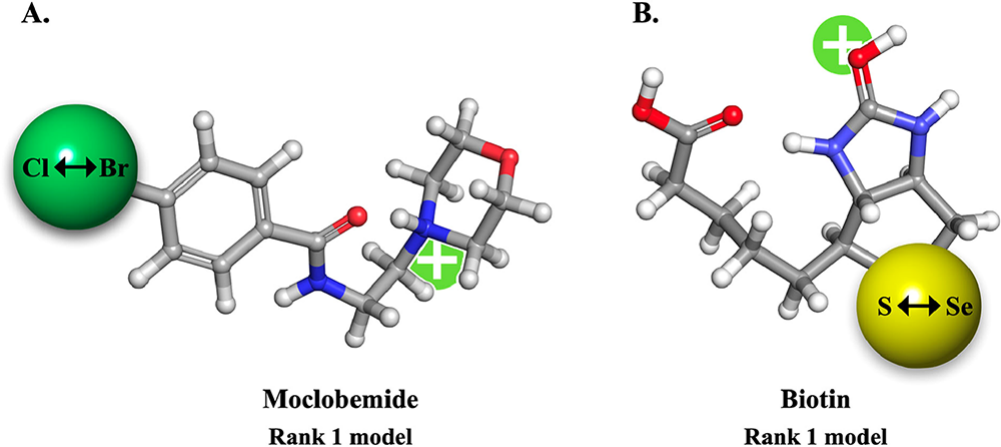
Equilibrium protonation models generated for biotin and moclobemide and their respective derivatized forms using the surrogacy method. The ensemble of conformations for moclobemide and biotin was geometry optimized at the D3-B3LYP/6-31G­(d,p) level. (A) Moclobemide and (B) biotin arrive at the same energy minimum charge site as their derivatives.

Moreover, the new RE values calculated from ANI-2x optimization were then used to rerank the protonation/deprotonation models that were initially ranked by GBT (for RE and mole fraction results, see Tables S3 and S4). Thus, the rankings that are outputted at this step are the final rankings, which make, for example, a *Rank* 1 model here the assigned major equilibrium charge state.

### Prediction Performance Assessment and Benchmarking Study

To assess the performance quality of SEER, we utilized test sets of 15 and 12 unseen systems for [M + H]+ and [M – H]–, respectively, and compared the equilibrium charge states predicted for the test set to the DFT ground truths. The DFT charge state ground truths were obtained by charge state modeling all readily titratable sites for the test set (refer to the workflow in Figure S1). The IM-MS experimental validity of the DFT ground truths was confirmed through achieving an experimental CCS with *a* ≤ 3% error. The same 27 systems in the test sets for [M + H]+ and [M – H]– were carried over and used for the benchmarking study. We elected to challenge SEER’s performance against CREST and Epik because they have uniquely different approaches for charge state determination, good efficiency, and are reasonably reliable. Similarly, the charge states predicted by CREST and Epik were compared to the DFT ground truths. Ideally, we want a top ranked model (i.e., *Rank 1*) assigned by any method to agree with the corresponding DFT ground truth charge state because it is the most energetically stable or the equilibrium charge state for a system, which in that case means that only a single protonation/deprotonation model is needed to correctly capture an experimental observation.

## Results and Discussion

To satisfactorily probe both the accuracy and generalizability of the SEER, we constructed the test sets using a diverse collection of small molecules, amino acids, oligopeptides, and nucleosides. As indicated earlier, we tested 15 and 12 new systems each for the [M + H]+ and [M – H]– ion mode, respectively, and we believe that this amount is sufficient to confidently draw a conclusion about SEER’s general performance and degree of interoperability. With that said, the largest biomolecules used in training are oligopeptides of four amino acid residues, and extrapolating a charge state for molecules beyond the training limit was not explored in the current work; however, we found that common protonation or deprotonation occurs at the peripheral or terminal loci, which then makes the effect of molecular size less of a factor. Furthermore, we want to emphasize that performance concerns for larger peptides are obviated because charge state increases as acidic or basic residues accumulate, which automatically render SEER inapplicable.

### Performance on Unseen Test Sets for the Negative and Positive Ion Modes

The results that established the DFT ground truths for the 27 systems in the test sets are shown in Figure 3A,B. The average IM-MS experimental agreement for both charge modes is ∼98% (or a CCS error of 1.6% for [M + H]+ and 1.3% for [M – H]−), with all systems achieving a CCS error of <3% (for detailed CCS performance results, see Tables S5 and S6), which qualifies them as valid standards to be used for experimental charge state confirmation in this work.

**Figure 3:**
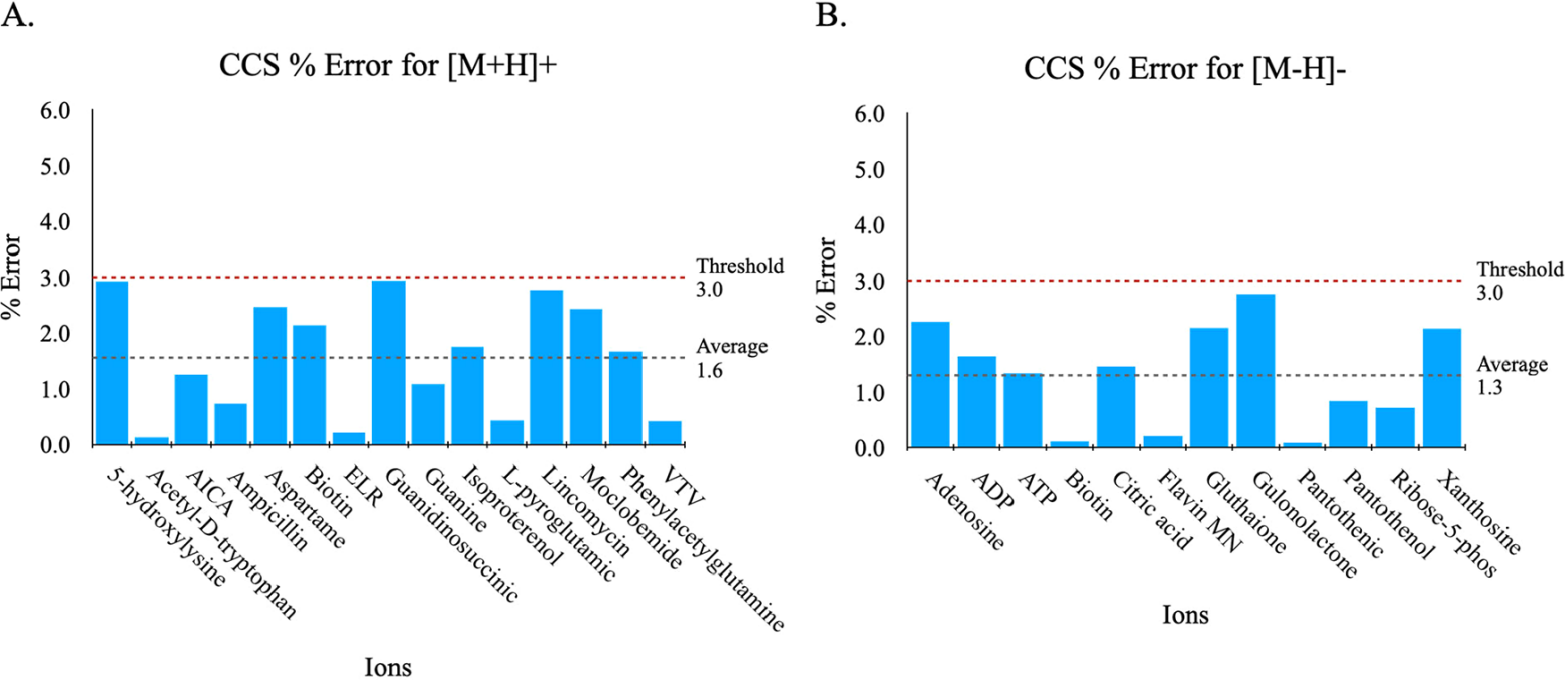
Validity of the DFT ground truth charge states. Experimental agreement of (A) D3BJ-B3LYP/6-31G­(d,p) for [M + H]+ and (B) D3BJ-B3LYP/6-31+G­(d,p) for [M – H]– as determined by CCS error performance. Note that CCS error of ≤ 3% is the threshold for acceptable accuracy.

For the performance evaluation step, 3D structures for the test set were generated from SMILE strings either by using Maestro^34^ (with the OPLS4 force field) or in the SEER platform using Open Babel^35^ with the following lines of Python code:
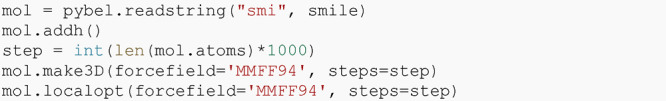


We implemented the MMFF94 force field to optimize the structures with the number of optimization steps customized to the number of atoms to account for differences in size and rotatable bonds. For [M + H]+, SEER demonstrated that it can successfully capture all experimental protomers (i.e., DFT ground truths) using less than two protonation models (mean ≈ 1.3) for the systems within this test set that have an average titratable site of ∼7 (Figure 4A). Similarly, 10 of 12 predicted equilibrium charge states that agree with experiment for the negative ion mode, [M – H], were assigned as *Rank* 1 by SEER (Figure 4B), which means that only two deprotonation models are essentially required to obtain the correct experimental charge states for systems in this test set. These results demonstrate that SEER is both generalizable and accurate at locating correct charge sites with very minimal variation in precision across systems.

**Figure 4:**
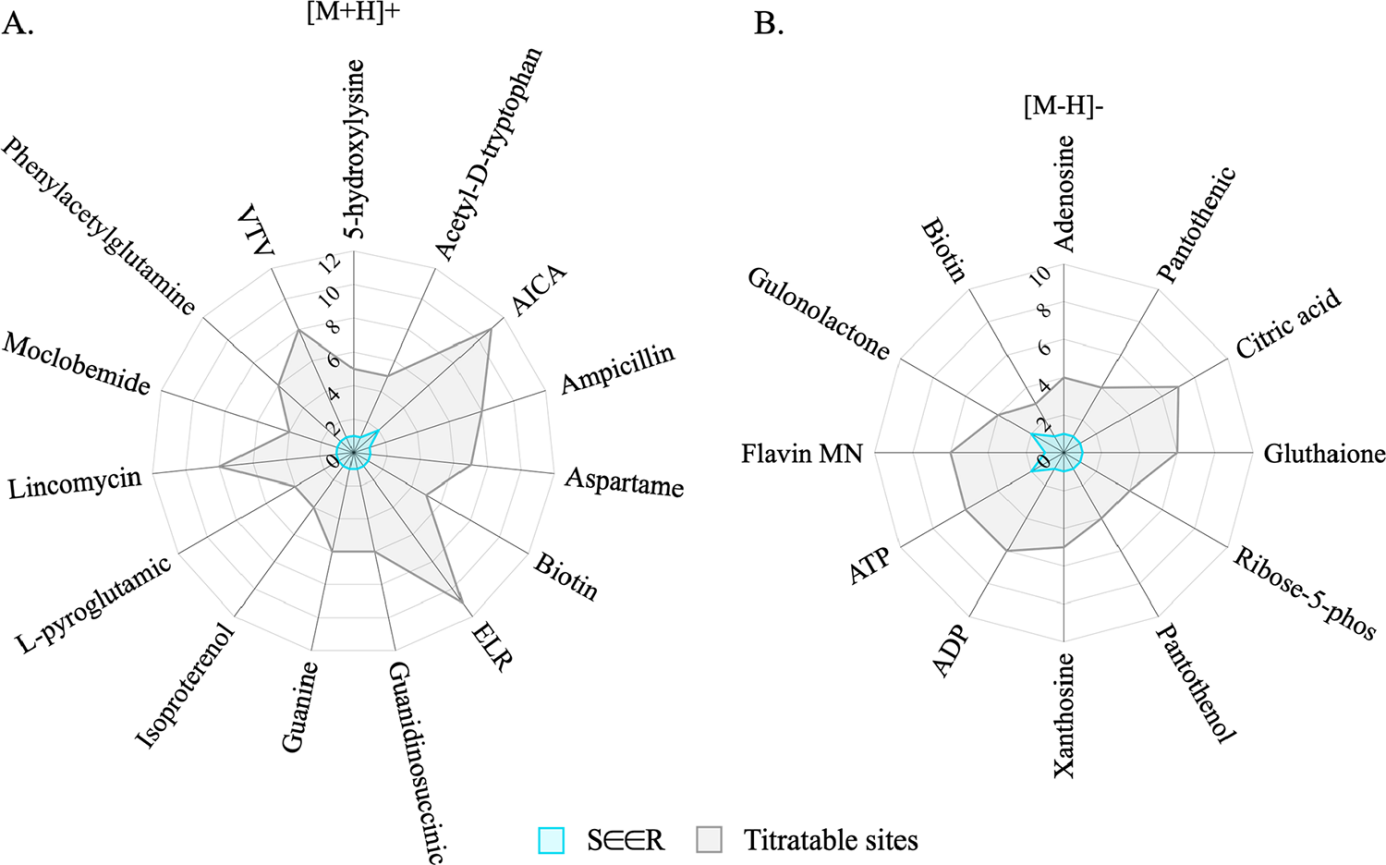
Number of titratable sites (gray) and protonation/deprotonation model(s) needed (blue) to successfully capture experimental charge states after using SEER for systems in the (A) [M + H]+ (protonated) test set and the (B) [M – H]– (deprotonated) test set.

Given that the goal of this work is to drastically reduce the overall modeling workload, we also probe the performance efficiency, or computational cost, of implementing SEER. Because molecules with different numbers of titratable sites may generate fewer or greater numbers of protonation/deprotonation models (i.e., enumerated charge sites), it is more appropriate to assess throughput on a model basis or runtime per model rather than on a runtime per system. Overall, the average runtime per model is 70 s for molecules in the [M + H]+ test set and 80 s for those in the [M – H]– test set (for more details, see Figure 5). We note that the most time-consuming step by far is the ANI-2x geometry optimization, which can take roughly >96% of the total job runtime. Moreover, the speed can be significantly improved by decreasing the number of optimization steps; however, we found that the value set (50 steps) is sufficient for a good balance of efficiency and quality of result.

**Figure 5:**
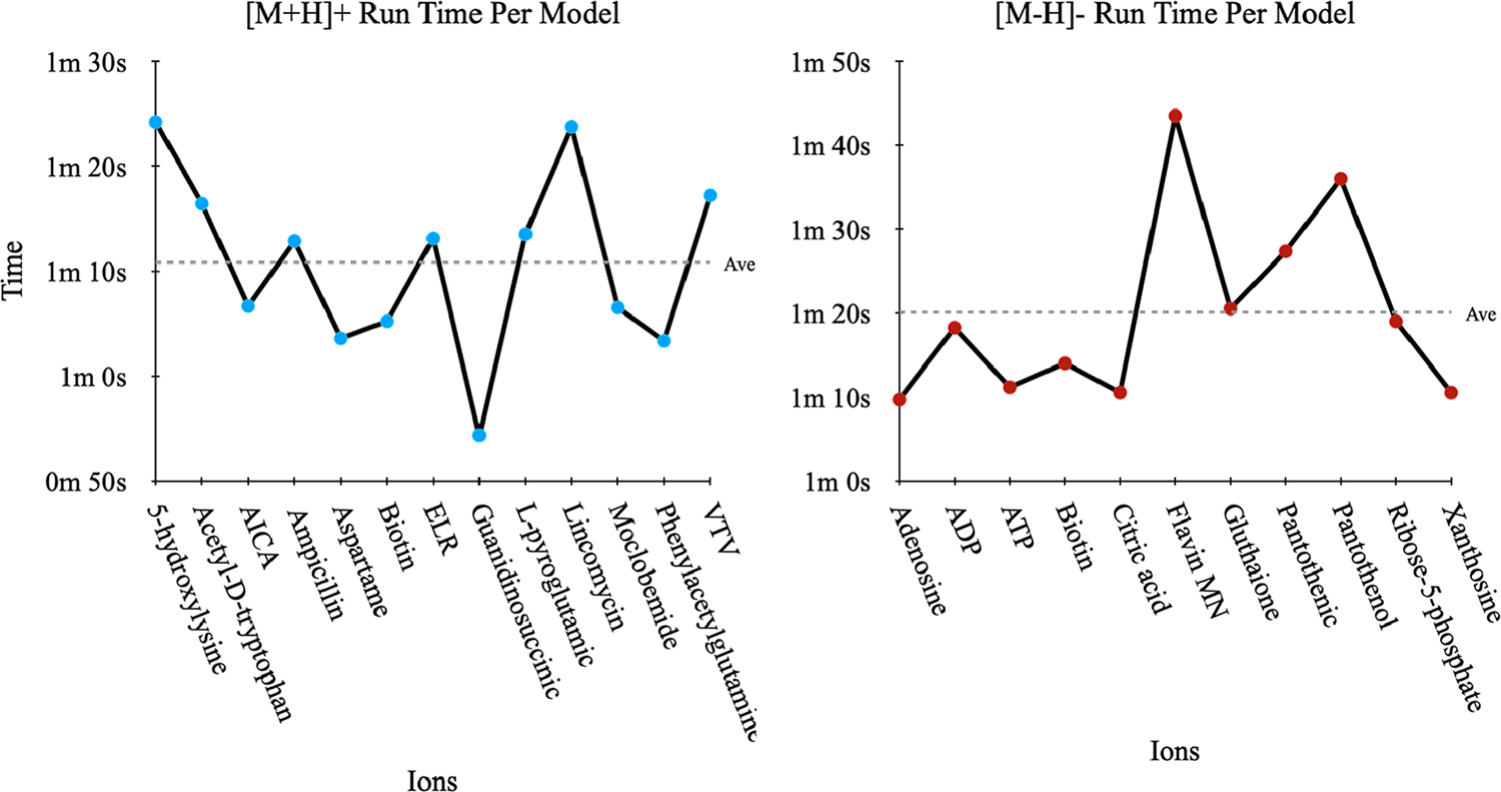
Processing or runtime for assigning charge state per model using SEER taken for the (A) [M + H]+ and (B) [M – H]+ test set. Depending on how many models are selected (autonomously), the total runtime may vary significantly from system to system. The time is in minutes and seconds. The energies for [M + H]+ and [M – H]– are ∼1 m 10 s and ∼1 m 20 s, respectively.

### Benchmarking and Performance Differentiation

For benchmarking, we first ran a general charge state similarity survey on 17 arbitrary new batches of molecules ranging in size and class to determine the degree of overlap between the predictions made by SEER, CREST, and Epik. To do this, we took the lowest relative energy for the protonated and deprotonated forms of the molecules obtained by SEER and compared them to CREST, Epik, and Epik/CREST. Figure 6 shows that *Rank* 1 charge states generated by SEER overlap 50 and 44% to the rank one candidates (rank is based on RE values) produced by CREST for the [M + H]+ and [M – H]– mode, respectively; whereas, overlaps of 38 and 69% are observed for rank one candidates (rank is based on p*K*_a_ values) generated by Epik; Altogether, SEER’s predictions for [M + H]+ and [M – H]– align 63 and 75%, respectively, to either CREST or Epik in selecting charge sites. Here, the results are intended to show only the agreement between the methods, while the validity of these predictions was, however, not verified for experimental accuracy.

**Figure 6:**
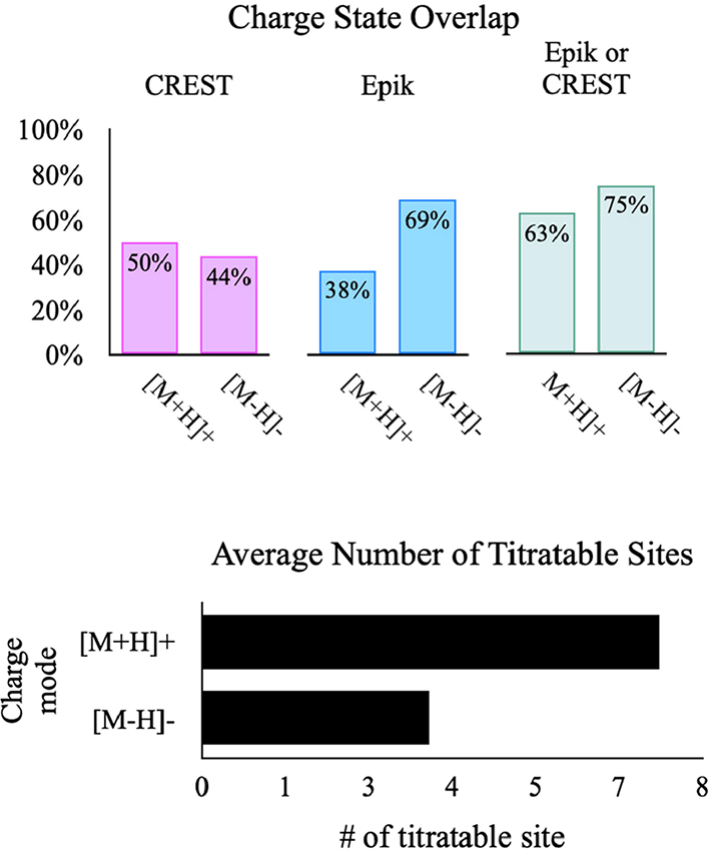
Overlap results of predicted top rank candidate charge states for 17 systems between SEER and CREST, Epik, or Epik/CREST (top). The average number of titratable sites for the same systems for [M + H]+ and [M – H]– modes (bottom).

Next, we ran CREST and Epik on the same test sets that we used to authenticate SEER’s performance on unseen systems. We mapped the predicted charge site results from the three methods on to the respective chemical structures. Figure 7A shows that for the [M + H]+ mode test set SEER proves to be more precise and accurate at capturing the correct experimental protomers. Both CREST and Epik are also accurate but less precise because for several systems (e.g., VTV, 5-hydroxylysine, aspartame, phenylacetylglutamine) they would require up to two and four protonation models, respectively, to correctly capture experiments. For the [M – H]– test set, SEER and Epik had similar performances (Figure 7B), with all experimental charge states correctly identified. On the other hand, CREST did not reproduce the DFT ground truth candidate charge states for glutathione and pantothenol.

**Figure 7:**
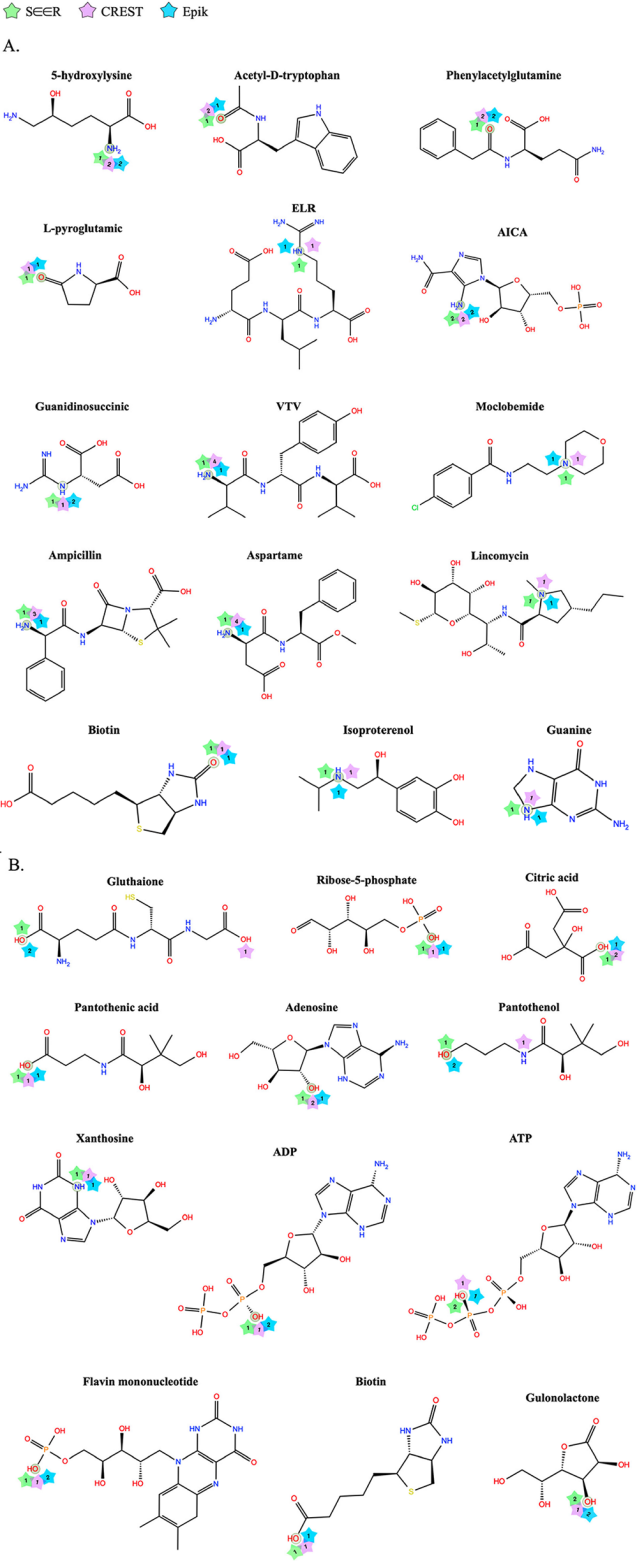
Benchmark results comparing SEER to CREST and Epik. The DFT ground truth atomic charge site for each system is shaded in gray and is the point of target for stars representing SEER (green), CREST (purple), and Epik (blue). The number in the star designates the relative energy rank of the predicted charge site, with rank 1 being the most favorable equilibrium gas-phase charge state predicted. The (A) [M + H]+ (protonated) test set contains 15 systems and (B) [M – H]– (deprotonated) test set contains 12.

In general, we have shown that SEER has been more consistent at assigning IM-MS experimentally relevant charge states to the highest ranked candidate (i.e., *Rank* 1) relative to CREST and Epik. We can quantify this overall precision with the use of confusion matrices that compare ground truth hit-and-miss for the three methods (Figure 8A). As shown in Figure 8A, the occurrence of correctly classified ground truth charge states (i.e., [M + H]+ and [M – H]−) as *Rank 1* is significantly higher for SEER. Moreover, any ranking assignment other than a *Rank 1* designation signifies that more than one protonation/deprotonation model would be required to capture an IM-MS experimentally viable charge state. According to Figure 8B,C, we can expect that users would ultimately depend on fewer models to confidently resolve a correct gas-phase structure with SEER. For example, in retrospect, to guarantee an above average success rate (>80%) in predicting the correct protomeric structures for the [M + H]+ test set, we would have needed up to four models for CREST, two for Epik, and one for SEER. Overall, we believe that SEER offers a performance advantage.

**Figure 8:**
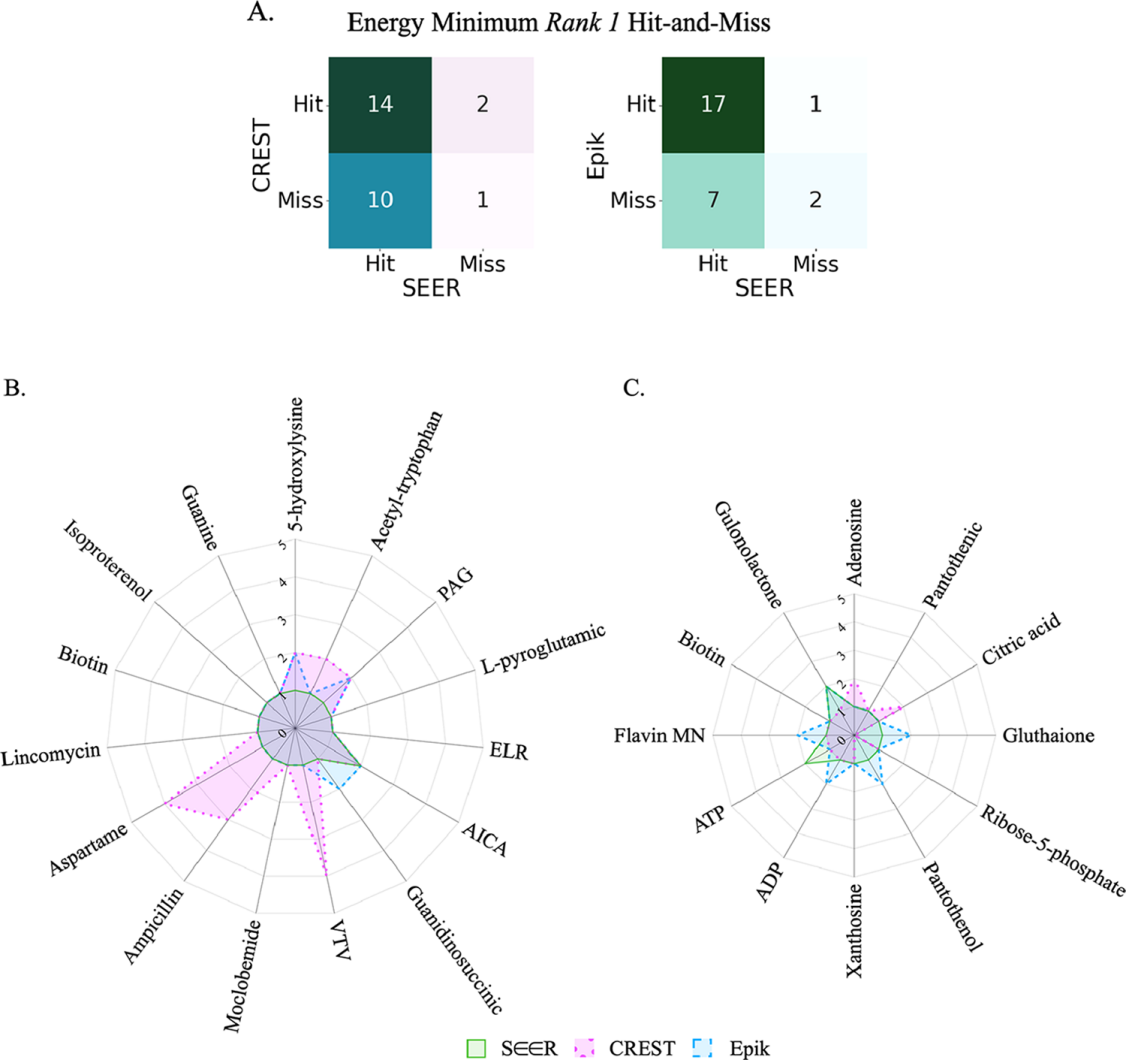
(A) Confusion matrix showing CREST or Epik ground truth *Rank 1* hit-and-miss alignment with SEER for 27 test systems. Retrospective number of protonation/deprotonation models that are required to capture the equilibrium charge state for B) [M + H]+ and C) [M – H]– test systems using SEER, CREST, or Epik.

### SEER User Guide

SEER is written in Python, and the service is readily deployable on the Google Colab platform. The only requirement, other than a Google account, is a user input chemical system that can be accepted as a SMILE or as an XYZ formatted file. Two options for charge state modeling are supported and available for selection from the pull-down menu: [M + H]+ and [M – H]–. Upon job completion, a TBD (to be determined) number of optimized and ranked models is partitioned into individual XYZ files and deposited into a directory named “/completed/[user molecule name]/”. Also, a job summary report containing information about tautomer energetics and gas-phase mole fraction is deposited into the same directory. The output folder containing the results can be accessed as a zip. The link to the SEER platform is accessible at https://github.com/mitkeng/SEER.

Currently, SEER supports only the generation of singly deprotonated or protonated ions. The training data exclusively pertain to common N and O titratable sites that are frequently observed in IM-MS experiments for biomolecules and biologically relevant small molecules, which, consequently, limits SEER’s ability to effectively recognize the protonation/deprotonation capability of other potentially titratable atom types like “othergens” (P, S, and Se). Another limitation is that SEER does not support deprotonation/protonation of carbon because there is very little experimental data available for proper training, and even with sufficient data, the relative abundance of carbon atoms in most molecules makes determining the exact charge-bearing carbon atom for use as a ground truth standard very challenging. We believe that these limitations, however, are not major disadvantages, since they are relevant to uncommon experimental situations. On the other hand, the benefits and advantages that SEER offers are

- High result TAT
- Unambiguous results
- Good generalizability
- Competitive accuracy
- No structural artifacts
- User-friendly interface
- Seamless workflow integration
- Open source

## Conclusions

There are few software tools that are reliable and readily accessible for modeling gas-phase chemical structure, especially one that is well-suited for resolving ion-mobility mass spectrometry results. We have demonstrated in prior studies that successful elucidation of an ion’s gas-phase equilibrium conformation for a given IM-MS derived CCS value is achievable with charge state modeling, conformation sampling, QM level processing, and CCS computation. However, the large computational expense involved in the QM step makes modeling molecules with many titratable sites time-consuming and even impractical. In response to this situation, we developed SEER to efficiently enumerate prototropic atoms to locate the equilibrium charge state or tautomer without resorting to the full QM treatment.

In this work, we reported that SEER performed well in capturing the DFT ground truth standards for systems in the test set. Moreover, SEER was able to capture the experimental charge states for both the negative mode, [M – H]–, and positive mode, [M + H]+, within two or fewer predicted charge state models (mean ≈ 1.1). When compared with CREST and Epik, we found that SEER’s performance is on par with their level of accuracy and even demonstrated better precision. Although the data set used to train the GBT model in SEER is relatively small, we believe that the addition of ANI-2x as a secondary model helps to improve the accuracy when new and unfamiliar molecules are encountered. Albeit there are current limitations to our software, we plan to add more data points to the training as we continue to process more chemical systems. All around, we believe that SEER provides a highly effective addition to a modeling workflow.

## Supplementary Material



## Data Availability

XYZ files for [M + H]+ and [M – H]– test set, ML models training data, and link to the SEER Google Colab notebook are accessible at https://github.com/mitkeng/seer

## Notes

Workflow for DFT ground truth procurement, feature importance analysis, hyperparameter setting, SEER output results, CCS validation results, and IM-MS experimental references (PDF)
