# Supplementary material for: State Ensemble Energy Recognition (SEER): A Hybrid Gas-Phase Molecular Charge State Predictor
Source: J Chem Inf Model. 2025 Jul 8;65(14):7507–15. doi: 10.1021/acs.jcim.5c00980 (PMC12308789; doi:10.1021/acs.jcim.5c00980)
Supplement: Supplementary file 1 [file ci5c00980_si_001.pdf]

# S ∈ ∈ R: A Hybrid Gas Phase Molecular Charge State Predictor

Mithony Keng and Kenneth M Merz, Jr.*

Department of Chemistry, Michigan State University,

East Lansing, Michigan 48824, United States

Department of Biochemistry and Molecular Biology, Michigan State University,

East Lansing, Michigan 48824, United States

*Corresponding Author: Kenneth M. Merz

*Corresponding Author Email: merz@chemistry.msu.edu

**Figure S1**. A schematic of the workflow to elucidate ion conformation and for establishing the DFT ground truth (IM-MS experimentally valid) charge state standard, which is used to confirm the validity of SEER's prediction. Both the energy minimum and ≤3% computed CCS error (against reference) requirements must be met to quality as a ground truth standard.

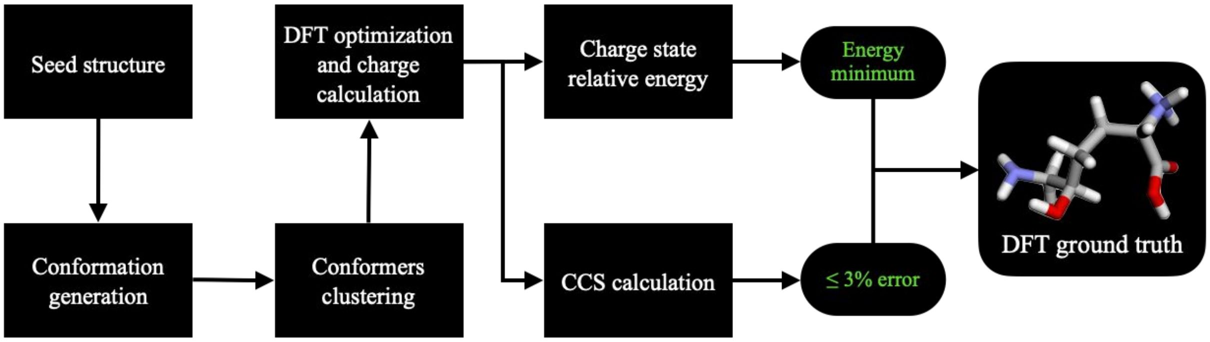


**Table S1**. Feature importance analysis. The mean increase in RMSE (MIR) is a measurement of prediction error with a permutative removal of a feature. The higher the MIR the greater the importance of a feature to the overall model performance.

| | Features | Mean Increase in RMSE |
|---|---|---|
| 1 | Molecular surface area | 17.870881 |
| 2 | Total oxygen | 10.230153 |
| 3 | COE distance | 8.593175 |
| 4 | COM↔COE distance | 7.217561 |
| 5 | Total nitrogen | 5.653583 |
| 6 | Interaction angle | 5.194336 |
| 7 | Atomic polarizability | 4.939976 |

**Table S2**. Fine-tune hyperparameter setting for the YDF GBT regression model. The "or" in setting represents conditional option that depends on whether [M+H]+ or [M-H]- is selected.

| | Hyperparameters | Setting |
|---|---|---|
| 1 | growing_strategy | BEST_FIRST_GLOBAL |
| 2 | max_depth | 8 or 10 |
| 3 | sampling_method | RANDOM |
| 4 | weights | COM_dist or None |
| 5 | split_axis | SPARSE_OBLIQUE |
| 6 | categorical_algorithm | RANDOM |

**Table S3**. Energy and charge state gas phase mole fraction results for protonated, [M+H]+, test set. Charge state model relative energy computed for SEER output charge model structure (ANI-2x geometry optimization and single point energy). Mole fraction is obtained from Boltzmann weighting the relative energy at 298 Kelvin.

**5-hydroxylysines**

| Test ID | Energy (Hartree) | Relative Energy (kcal/mol) | Mole Fraction |
|---|---|---|---|
| Model Rank1 | -572.6789 | 0 | 1 |
| Model Rank2 | -572.6484 | 19.15 | 0 |

**N-Acetyl-D-tryptophan**

| Test ID | Energy (Hartree) | Relative Energy (kcal/mol) | Mole Fraction |
|---|---|---|---|
| Model Rank1 | -839.3858 | 0 | 1 |
| Model Rank2 | -839.3732 | 7.93 | 0 |

**Phenylacetylglutamine**

| Test ID | Energy (Hartree) | Relative Energy (kcal/mol) | Mole Fraction |
|---|---|---|---|
| Model Rank1 | -915.8296 | 0 | 0.99 |
| Model Rank2 | -915.8254 | 2.63 | 0.01 |
| Model Rank3 | -915.8124 | 10.79 | 0 |
| Model Rank4 | -915.8073 | 14.02 | 0 |

**L-pyroglutamic**

| Test ID | Energy (Hartree) | Relative Energy (kcal/mol) | Mole Fraction |
|---|---|---|---|
| Model Rank1 | -475.6605 | 0 | 1 |
| Model Rank2 | -475.6546 | 3.71 | 0 |

**ELR**

| Test ID | Energy (Hartree) | Relative Energy (kcal/mol) | Mole Fraction |
|---|---|---|---|
| Model Rank1 | -1447.2754 | 0 | 1 |
| Model Rank2 | -1447.2350 | 25.36 | 0 |
| Model Rank3 | -1447.2284 | 29.51 | 0 |
| Model Rank4 | -1447.2146 | 38.19 | 0 |

**AIC-ribonucleotide**

| Test ID | Energy (Hartree) | Rel. Energy (kcal/mol) | Mole Fraction |
|---|---|---|---|
| Model Rank1 | -1571.2032 | 0 | 1 |
| Model Rank2 | -1571.1880 | 9.54 | 0 |
| Model Rank3 | -1571.1865 | 10.49 | 0 |
| Model Rank4 | -1571.1861 | 10.7 | 0 |
| Model Rank5 | -1571.1777 | 15.99 | 0 |
| Model Rank6 | -1571.1691 | 21.39 | 0 |
| Model Rank7 | -1571.1648 | 24.05 | 0 |
| Model Rank8 | -1571.1642 | 24.48 | 0 |
| Model Rank9 | -1571.1572 | 28.86 | 0 |
| Model Rank10 | -1571.1553 | 30.01 | 0 |
| Model Rank11 | -1571.1518 | 32.26 | 0 |

**Guanidinosuccinic**

| Test ID | Energy (Hartree) | Relative Energy (kcal/mol) | Mole Fraction |
|---|---|---|---|
| Model Rank1 | -661.5829 | 0 | 1 |
| Model Rank2 | -661.5401 | 26.88 | 0 |
| Model Rank3 | -661.4962 | 54.42 | 0 |

**VTV**

| Test ID | Energy (Hartree) | Relative Energy (kcal/mol) | Mole Fraction |
|---|---|---|---|
| Model Rank1 | -1282.1878 | 0 | 0.54 |
| Model Rank2 | -1282.1877 | 0.1 | 0.46 |
| Model Rank3 | -1282.1751 | 8 | 0 |

**Moclobemide**

| Test ID | Energy (Hartree) | Relative Energy (kcal/mol) | Mole Fraction |
|---|---|---|---|
| Model Rank1 | -1226.1338 | 0 | 1 |
| Model Rank2 | -1226.1281 | 3.61 | 0 |
| Model Rank3 | -1226.1015 | 20.29 | 0 |
| Model Rank4 | -1226.0939 | 25.06 | 0 |

**Ampicillin**

| Test ID | Energy (Hartree) | Relative Energy (kcal/mol) | Mole Fraction |
|---|---|---|---|
| Model Rank1 | -1484.7966 | 0 | 1 |

| | | | |
|---|---|---|---|
| Model Rank2 | -1484.7789 | 11.15 | 0 |
| Model Rank3 | -1484.7739 | 14.27 | 0 |
| Model Rank4 | -1484.7716 | 15.71 | 0 |
| Model Rank5 | -1484.7635 | 20.79 | 0 |
| Model Rank6 | -1484.7507 | 28.84 | 0 |
| Model Rank7 | -1484.7461 | 31.69 | 0 |

**Aspartame**

| Test ID | Energy (Hartree) | Relative Energy (kcal/mol) | Mole Fraction |
|---|---|---|---|
| Model Rank1 | -1030.3348 | 0 | 1 |
| Model Rank2 | -1030.3004 | 21.59 | 0 |

**Lincomycin**

| Test ID | Energy (Hartree) | Relative Energy (kcal/mol) | Mole Fraction |
|---|---|---|---|
| Model Rank1 | -1665.6498 | 0 | 1 |
| Model Rank2 | -1665.6411 | 5.48 | 0 |
| Model Rank3 | -1665.6334 | 10.31 | 0 |
| Model Rank4 | -1665.6299 | 12.48 | 0 |
| Model Rank5 | -1665.6234 | 16.6 | 0 |
| Model Rank6 | -1665.6222 | 17.33 | 0 |
| Model Rank7 | -1665.6175 | 20.28 | 0 |

**Table S4**. Energy and charge state gas phase mole fraction results for deprotonated, [M-H]-, test set. Model relative energy computed for SEER output charge model structure (ANI-2x geometry optimization and single point energy). Mole fraction is obtained from Boltzmann weighting the relative energy at 298 Kelvin.

**Glutathione**

| Test ID | Energy (Hartree) | Relative Energy (kcal/mol) | Mole Fraction |
|---|---|---|---|
| Model Rank1 | -1404.2786 | 0 | 0.98 |
| Model Rank2 | -1404.2748 | 2.4 | 0.02 |

**Ribose-5-phosphate**

| Test ID | Energy (Hartree) | Relative Energy (kcal/mol) | Mole Fraction |
|---|---|---|---|
| Model Rank1 | -1196.1895 | 0 | 0.86 |
| Model Rank2 | -1196.1878 | 1.05 | 0.14 |
| Model Rank3 | -1196.1558 | 21.14 | 0 |

| | | | |
|---|---|---|---|
| Model Rank4 | -1196.155 | 21.63 | 0 |

**Citric acid**

| Test ID | Energy (Hartree) | Relative Energy (kcal/mol) | Mole Fraction |
|---|---|---|---|
| Model Rank1 | -759.2747 | 0 | 1 |
| Model Rank2 | -759.2644 | 6.51 | 0 |
| Model Rank3 | -759.2441 | 19.23 | 0 |

**Pantothenic acid**

| Test ID | Energy (Hartree) | Relative Energy (kcal/mol) | Mole Fraction |
|---|---|---|---|
| Model Rank1 | -783.2884 | 0 | 1 |

**Adenosine**

| Test ID | Energy (Hartree) | Relative Energy (kcal/mol) | Mole Fraction |
|---|---|---|---|
| Model Rank1 | -962.6783 | 0 | 0.96 |
| Model Rank2 | -962.6752 | 1.96 | 0.04 |
| Model Rank3 | -962.6709 | 4.68 | 0 |
| Model Rank4 | -962.662 | 10.22 | 0 |

**Xanthosine**

| Test ID | Energy (Hartree) | Relative Energy (kcal/mol) | Mole Fraction |
|---|---|---|---|
| Model Rank1 | -1057.7764 | 0 | 1 |
| Model Rank2 | -1057.7543 | 13.89 | 0 |

**ADP**

| Test ID | Energy (Hartree) | Relative Energy (kcal/mol) | Mole Fraction |
|---|---|---|---|
| Model Rank1 | -2211.3491 | 0 | 1 |
| Model Rank2 | -2211.3257 | 14.71 | 0 |
| Model Rank3 | -2211.3215 | 17.33 | 0 |
| Model Rank4 | -2211.3057 | 27.22 | 0 |

**Panthenol**

| Test ID | Energy (Hartree) | Rel. Energy (kcal/mol) | Mole Fraction |
|---|---|---|---|
| Model Rank1 | -709.2282 | 0 | 1 |

**Table S5.** Experimental agreeableness of equilibrium charge states for the established ground truth [M-H]- test set according to CCS performance.

| Molecular anion | Computed CCS[a] | Experiment CCS | % CCS Error | ref |
|---|---|---|---|---|
| Adenosine | 165.88 | 162.13 | 2.31 | 1 |
| Pantothenic acid | 146.74 | 146.6 | 0.10 | 2 |
| Citric acid | 131.41 | 129.49 | 1.48 | 1 |
| Glutathione | 167.83 | 164.24 | 2.19 | 1 |
| Ribose-5-phosphate | 140.65 | 141.66 | 0.71 | 1 |
| Pantothenol | 140.31 | 141.49 | 0.83 | 3 |
| Xanthosine | 164.87 | 161.36 | 2.18 | 1 |
| ADP | 189.97 | 186.87 | 1.66 | 1 |
| ATP | 201.56 | 198.86 | 1.36 | 1 |
| Flavin mononucleotide | 201.20 | 200.77 | 0.21 | 1 |

**Table S6.** Experimental agreeableness of equilibrium charge states for the established ground truth [M+H]+ test set according to CCS performance.

| Molecular cation | Computed CCS[a] | Experiment CCS | % CCS error | ref |
|---|---|---|---|---|
| 5-hydroxylysine | 130.20 | 134.00 | 2.84 | 2 |
| Acetyl-D-tryptophan | 155.88 | 155.67 | 0.13 | 1 |
| Phenylacetylglutamine | 156.11 | 158.70 | 1.63 | 3 |
| L-pyroglutamic | 120.49 | 121.00 | 0.42 | 4 |
| ELR | 199.88 | 200.30 | 0.21 | 5 |
| AICA | 173.72 | 171.60 | 1.24 | 3 |
| Guanidinosuccinic | 133.00 | 136.80 | 2.85 | 2 |
| VTV | 192.10 | 191.30 | 0.42 | 5, 6 |
| Moclobemide | 159.03 | 162.90 | 2.38 | 7 |
| Ampicillin | 182.62 | 186.75 | 2.21 | 8 |
| Aspartame | 165.53 | 169.60 | 2.40 | 3 |
| Lincomycin | 194.50 | 199.90 ± 2.6 | 2.70 | 9, 10 |

## REFERENCE

(1) Zheng, X.; Aly, N. A.; Zhou, Y.; Dupuis, K. T.; Bilbao, A.; Paurus, Vanessa L.; Orton, D. J.; Wilson, R.; Payne, S. H.; Smith, R. D.; et al. A structural examination and collision cross section database for over 500 metabolites and xenobiotics using drift tube ion mobility spectrometry. *Chemical Science* **2017**, *8* (11), 7724-7736, 10.1039/C7SC03464D. DOI: 10.1039/C7SC03464D.
(2) Nichols, C. M.; Dodds, J. N.; Rose, B. S.; Picache, J. A.; Morris, C. B.; Codreanu, S. G.; May, J. C.; Sherrod, S. D.; McLean, J. A. Untargeted Molecular Discovery in Primary Metabolism: Collision Cross Section as a Molecular Descriptor in Ion Mobility-Mass Spectrometry. *Analytical Chemistry* **2018**, *90* (24), 14484-14492. DOI: 10.1021/acs.analchem.8b04322.
(3) Zhou, Z.; Shen, X.; Tu, J.; Zhu, Z.-J. Large-Scale Prediction of Collision Cross-Section Values for Metabolites in Ion Mobility-Mass Spectrometry. *Analytical Chemistry* **2016**, *88* (22), 11084-11091. DOI: 10.1021/acs.analchem.6b03091.
(4) Paglia, G.; Williams, J. P.; Menikarachchi, L.; Thompson, J. W.; Tyldesley-Worster, R.; Halldórsson, S.; Rolfsson, O.; Moseley, A.; Grant, D.; Langridge, J.; et al. Ion Mobility Derived Collision Cross Sections to Support Metabolomics Applications. *Analytical Chemistry* **2014**, *86* (8), 3985-3993. DOI: 10.1021/ac500405x.
(5) Picache, J. A.; Rose, B. S.; Balinski, A.; Leaptrot, Katrina L.; Sherrod, S. D.; May, J. C.; McLean, J. A. Collision cross section compendium to annotate and predict multi-omic compound identities. *Chemical Science* **2019**, *10* (4), 983-993, 10.1039/C8SC04396E. DOI: 10.1039/C8SC04396E.
(6) Nichols, C. M.; May, J. C.; Sherrod, S. D.; McLean, J. A. Automated flow injection method for the high precision determination of drift tube ion mobility collision cross sections. *Analyst* **2018**, *143* (7), 1556-1559, 10.1039/C8AN00056E. DOI: 10.1039/C8AN00056E.
(7) Lian, R.; Zhang, F.; Zhang, Y.; Wu, Z.; Ye, H.; Ni, C.; Lv, X.; Guo, Y. Ion mobility derived collision cross section as an additional measure to support the rapid analysis of abused drugs and toxic compounds using electrospray ion mobility time-of-flight mass spectrometry. *Analytical Methods* **2018**, *10* (7), 749-756, 10.1039/C7AY02808C. DOI: 10.1039/C7AY02808C.
(8) Mohammed Taha, H.; Aalizadeh, R.; Alygizakis, N.; Antignac, J.-P.; Arp, H. P. H.; Bade, R.; Baker, N.; Belova, L.; Bijlsma, L.; Bolton, E. E.; et al. The NORMAN Suspect List Exchange (NORMAN-SLE): facilitating European and worldwide collaboration on suspect screening in high resolution mass spectrometry. *Environmental Sciences Europe* **2022**, *34* (1), 104. DOI: 10.1186/s12302-022-00680-6.
(9) Bijlsma, L.; Bade, R.; Celma, A.; Mullin, L.; Cleland, G.; Stead, S.; Hernandez, F.; Sancho, J. V. Prediction of Collision Cross-Section Values for Small Molecules: Application to Pesticide Residue Analysis. *Analytical Chemistry* **2017**, *89* (12), 6583-6589. DOI: 10.1021/acs.analchem.7b00741.
(10) Ross, D. H.; Seguin, R. P.; Krinsky, A. M.; Xu, L. High-Throughput Measurement and Machine Learning-Based Prediction of Collision Cross Sections for Drugs and Drug Metabolites. *Journal of the American Society for Mass Spectrometry* **2022**, *33* (6), 1061-1072. DOI: 10.1021/jasms.2c00111.
